# Gene expression analysis of epithelial-mesenchymal transition markers in oral submucous fibrosis fibroblasts treated with arecoline

**DOI:** 10.6026/973206300213871

**Published:** 2025-10-31

**Authors:** Jigar P Thakkar, Gopal Srivastava, Khushali Shah, Vipin Arora, Mony Behera, Sameer Gupta

**Affiliations:** 1 Department of Dentistry, GMERS Medical College, Vadnagar, Gujarat, India; 2 Department of Oral Medicine and Radiology, Government Dental College, Thiruvananthapuram, Kerala, India; 3 Department of Oral and Maxillofacial Pathology, K.M. Shah Dental College and Hospital, Sumandeep Vidyapeeth Deemed to be university, Vadodara, Gujarat, India; 4 Department of Restorative Dental Sciences, Faculty of Dentistry, Taif University, Taif, Kingdom of Saudi Arabia; 5 Department of Oral Medicine and Radiology, Kalinga Institute of Dental Sciences (KIDS), Kalinga Institute of Industrial Technology (KIIT) Deemed to be University, Bhubaneswar-751024, Odisha, India; 6 Department of Oral & Maxillofacial Surgery, Inderprastha Dental College and Hospital, Sahibabad, Ghaziabad, Uttar Pradesh, India

**Keywords:** Oral submucous fibrosis, epithelial-mesenchymal transition, arecoline, curcumin, EGCG, fibroblasts

## Abstract

Oral submucous fibrosis (OSF), a potentially malignant disorder, is strongly linked to areca nut consumption, with arecoline inducing epithelial-mesenchymal transition (EMT) in fibroblasts. Therfeore, it is of interest to investigate EMT marker expression in OSF fibroblasts exposed to arecoline and assessed the inhibitory effects of curcumin and epigallocatechin-3-gallate (EGCG). Primary fibroblasts from OSF and normal mucosa were treated and EMT markers were analyzed by RT-qPCR, Western blotting and wound healing assays. Arecoline decreased E-cadherin and increased N-cadherin, vimentin, α-SMA, Snail, Twist and migration rate, whereas co-treatment with curcumin or EGCG significantly reversed these effects, with curcumin showing greater efficacy. Thus, we show that curcumin and EGCG inhibit arecoline-induced EMT and may serve as potential therapeutic agents in OSF management.

## Background:

Oral submucous fibrosis (OSF) represents a chronic, progressive and potentially malignant disorder affecting the oral cavity, characterized by inflammation and progressive fibrosis of the submucosal tissues [1]. The condition predominantly affects populations in South and Southeast Asia, with prevalence rates ranging from 0.2% to 2.3% in various Indian states [2]. The primary etiological factor associated with OSF is the habitual chewing of areca nut, either alone or in combination with tobacco products [3]. Arecoline, the principal alkaloid constituent of areca nut, has been extensively implicated in the pathogenesis of OSF through multiple mechanisms including enhanced collagen synthesis, reduced collagen degradation and induction of fibroblast proliferation [4]. Recent studies have demonstrated that arecoline can trigger epithelial-mesenchymal transition (EMT), a fundamental biological process wherein epithelial cells lose their characteristic features and acquire mesenchymal properties [5]. This transition is marked by the loss of epithelial markers such as E-cadherin and the acquisition of mesenchymal markers including N-cadherin, vimentin and α-smooth muscle actin (α-SMA) [6]. The EMT process in OSF contributes significantly to the progressive nature of the disease and its potential for malignant transformation [7]. Understanding the molecular mechanisms underlying arecoline-induced EMT in oral fibroblasts is crucial for developing targeted therapeutic interventions. Several natural compounds have shown promise in modulating EMT processes in various fibrotic conditions [8]. Among these, curcumin and epigallocatechin-3-gallate (EGCG) have demonstrated anti-fibrotic properties through their ability to interfere with TGF-β signaling pathways and oxidative stress mechanisms [9]. Despite the growing understanding of EMT in fibrotic diseases, comprehensive analysis of EMT marker expression in OSF fibroblasts following arecoline exposure and the potential inhibitory effects of natural compounds remain inadequately explored. Previous studies have primarily focused on individual markers or pathways, lacking a holistic approach to understanding the EMT process in OSF [10]. Furthermore, the comparative efficacy of different natural compounds in reversing arecoline-induced EMT has not been systematically evaluated. Therefore, it is of interest to comprehensively analyze the expression profile of key EMT markers in primary fibroblasts isolated from OSF tissues following arecoline treatment and to evaluate the inhibitory potential of curcumin and EGCG on arecoline-induced EMT processes.

## Materials and Methods:

## Study design and sample collection:

This experimental study was conducted using primary fibroblast cultures derived from human oral tissue samples. Tissue specimens were obtained from 15 patients diagnosed with OSF (study group) and 10 healthy individuals undergoing minor oral surgical procedures (control group).

## Inclusion and exclusion criteria:

Inclusion criteria for the OSF group included: clinically and histopathologically confirmed OSF diagnosis, age 25-50 years and history of areca nut chewing for at least 5 years. Exclusion criteria comprised: presence of oral malignancy, systemic diseases affecting wound healing, recent corticosteroid therapy (within 3 months) and pregnancy or lactation.

## Primary fibroblast isolation and culture:

Tissue samples were collected in sterile phosphate-buffered saline containing antibiotics. Fibroblasts were isolated using the explant culture method. Briefly, tissues were minced into 1-2 mm^3^ pieces and placed in culture dishes with Dulbecco's Modified Eagle Medium (DMEM) supplemented with 20% fetal bovine serum, 100 U/ml penicillin and 100 µg/ml streptomycin. Cultures were maintained at 37°C in a humidified atmosphere with 5% CO_2_. Fibroblasts between passages 3-6 were used for experiments.

## Treatment protocol:

Cells were seeded at a density of 5x10^4^ cells/cm^2^ and allowed to reach 70-80% confluence. Treatment groups included: (1) Control (vehicle only), (2) Arecoline (50 µg/ml), (3) Arecoline + Curcumin (50 µg/ml + 20 µM), (4) Arecoline + EGCG (50 µg/ml + 50 µM), (5) Curcumin alone (20 µM) and (6) EGCG alone (50 µM). Treatments were administered for 48 hours with media change at 24 hours.

## RNA extraction and quantitative RT-PCR:

Total RNA was extracted using TRIzol reagent following manufacturer's protocol. RNA quality was assessed using spectrophotometry (A260/A280 ratio >1.8). cDNA synthesis was performed using 2 µg total RNA with reverse transcriptase kit. Quantitative PCR was conducted using SYBR Green master mix with specific primers for E-cadherin, N-cadherin, vimentin, α-SMA, Snail, Twist and GAPDH (housekeeping gene). Relative expression was calculated using the 2^(-ΔΔCt) method.

## Western blot analysis:

Cells were lysed in RIPA buffer containing protease inhibitors. Protein concentration was determined using BCA assay. Equal amounts of protein (30 µg) were separated on 10% SDS-PAGE gels and transferred to PVDF membranes. Membranes were blocked with 5% non-fat milk and incubated with primary antibodies overnight at 4°C, followed by HRP-conjugated secondary antibodies. Protein bands were visualized using enhanced chemiluminescence and quantified using ImageJ software.

## Cell migration assay:

Wound healing assay was performed by creating a uniform scratch in confluent cell monolayers using a sterile pipette tip. Cells were washed and treated with respective compounds in serum-reduced media (2% FBS). Images were captured at 0, 24 and 48 hours using phase-contrast microscopy. Wound closure was calculated as percentage of initial wound area.

## Statistical analysis:

Data were expressed as mean ± standard deviation from three independent experiments performed in triplicate. Statistical analysis was performed using GraphPad Prism 9.0. One-way ANOVA followed by Tukey's post-hoc test was used for multiple group comparisons. Student's t-test was used for two-group comparisons. P-values <0.05 were considered statistically significant.

## Results:

Primary fibroblasts isolated from both OSF and normal tissues exhibited typical spindle-shaped morphology. Immunofluorescence staining confirmed positive expression of vimentin (98.4% ± 1.2%) and negative expression of cytokeratin, confirming their mesenchymal phenotype. OSF-derived fibroblasts showed enhanced proliferation rate compared to normal fibroblasts (doubling time: 28.3 ± 3.1 hours vs. 36.7 ± 4.2 hours, p<0.01). Arecoline treatment significantly altered the expression profile of EMT markers in OSF fibroblasts. E-cadherin mRNA expression decreased to 0.32 ± 0.08-fold compared to control (p<0.001), while mesenchymal markers showed substantial upregulation: N-cadherin (3.24 ± 0.41-fold), vimentin (2.87 ± 0.35-fold) and α-SMA (4.12 ± 0.52-fold) (all p<0.001 vs. control). Transcription factors Snail and Twist were upregulated by 2.56 ± 0.31-fold and 2.13 ± 0.28-fold, respectively (p<0.001) (Table 1 - see PDF). Western blot analysis corroborated the mRNA findings. Arecoline treatment resulted in 68.4% ± 7.2% reduction in E-cadherin protein levels (p<0.001), while N-cadherin, vimentin and α-SMA protein levels increased by 287% ± 32%, 254% ± 28% and 378% ± 41%, respectively (all p<0.001). Co-treatment with curcumin or EGCG significantly attenuated these changes, with curcumin demonstrating superior efficacy in preserving E-cadherin expression and limiting mesenchymal marker upregulation (Table 2 - see PDF). Arecoline treatment significantly enhanced fibroblast migration capacity. At 48 hours, arecoline-treated cells showed 78.4% ± 9.2% wound closure compared to 42.3% ± 5.8% in control cells (p<0.001). Co-treatment with curcumin reduced migration to 24.3% ± 5.1% wound closure, while EGCG co-treatment resulted in 31.7% ± 6.4% closure (both p<0.001 vs. arecoline alone). Neither curcumin nor EGCG alone significantly affected baseline migration rates (Table 3 - see PDF).

## Discussion:

The present study provides comprehensive evidence that arecoline induces EMT in OSF-derived fibroblasts, characterized by downregulation of epithelial markers and upregulation of mesenchymal markers, which can be effectively attenuated by natural compounds curcumin and EGCG. These findings have significant implications for understanding OSF pathogenesis and developing therapeutic interventions. Our results demonstrating arecoline-induced downregulation of E-cadherin and upregulation of N-cadherin, vimentin and α-SMA are consistent with previous studies investigating EMT in fibrotic disorders [11]. The magnitude of change observed in our study, particularly the 4-fold increase in α-SMA expression, suggests a robust myofibroblast transformation, which is a hallmark of fibrotic progression [12]. The concurrent upregulation of transcription factors Snail and Twist indicates activation of the classical EMT regulatory pathway, as these factors directly repress E-cadherin transcription [13]. The enhanced migration capacity of arecoline-treated fibroblasts, demonstrated by a nearly 2-fold increase in wound closure rate, correlates with the mesenchymal phenotype acquisition. This finding aligns with studies showing that EMT confers increased motility and invasive properties to transformed cells [14]. The migration data, combined with molecular marker analysis, provides functional validation of the EMT process in our model system. The inhibitory effects of curcumin on arecoline-induced EMT observed in our study extend previous findings on its anti-fibrotic properties [15]. Curcumin's superior efficacy compared to EGCG in preserving E-cadherin expression and limiting mesenchymal marker upregulation may be attributed to its broader spectrum of molecular targets, including NF-κB, TGF-β/Smad and PI3K/Akt pathways [16]. The ability of curcumin to reduce cell migration below baseline levels when combined with arecoline suggests potential interference with multiple signaling cascades involved in cell motility [17].

EGCG, while less potent than curcumin in our model, still demonstrate significant protective effects against arecoline-induced EMT. Previous studies have shown that EGCG can inhibit TGF-β1-induced EMT through suppression of Smad2/3 phosphorylation and modulation of reactive oxygen species [18]. The differential efficacy between curcumin and EGCG observed in our study may reflect differences in their bioavailability, cellular uptake, or specific molecular targets in the context of arecoline exposure [19]. The clinical relevance of our findings is underscored by the high prevalence of OSF in populations with areca nut chewing habits and the limited therapeutic options currently available [20]. The demonstration that natural compounds can effectively counteract arecoline-induced EMT suggests potential for dietary or pharmacological interventions. However, translation to clinical practice requires consideration of bioavailability issues and optimal delivery methods for these compounds [21]. Our study also revealed interesting baseline differences between OSF-derived and normal fibroblasts, with OSF fibroblasts showing enhanced proliferation rates. This observation suggests that chronic arecoline exposure may induce persistent phenotypic changes in oral fibroblasts, potentially through epigenetic modifications [22]. This finding warrants further investigation into the reversibility of fibroblast activation in OSF and the potential for targeting resident fibroblast populations in therapeutic strategies. The comprehensive analysis of multiple EMT markers at both mRNA and protein levels strengthens the validity of our findings. The concordance between gene expression and protein data indicates that the observed changes reflect genuine biological alterations rather than transient transcriptional fluctuations [23]. Furthermore, the dose-dependent responses and the lack of significant effects with curcumin or EGCG alone support the specificity of their inhibitory actions against arecoline-induced changes. Several limitations of this study should be acknowledged. First, the in vitro model, while providing controlled experimental conditions, may not fully recapitulate the complex microenvironment of OSF tissues [24]. Second, the relatively short treatment duration (48 hours) may not reflect the chronic exposure patterns seen in OSF patients. Third, we focused on selected EMT markers and did not investigate all potential signaling pathways involved in the process [25]. Future research directions should include investigation of the specific molecular mechanisms underlying the protective effects of curcumin and EGCG, evaluation of combination therapies and assessment of these compounds in animal models of OSF [26]. Additionally, exploring the role of other EMT-related processes such as basement membrane degradation and extracellular matrix remodeling would provide a more complete understanding of OSF pathogenesis [27].

## Conclusion:

Arecoline induces a robust EMT program in OSF-derived fibroblasts, characterized by loss of epithelial markers, gain of mesenchymal markers and enhanced migratory capacity. Both curcumin and EGCG effectively attenuate these changes, with curcumin showing superior efficacy. Data provide mechanistic insights into OSF pathogenesis and suggest that targeting EMT through natural compounds may represent a viable therapeutic strategy for OSF management. Further research is warranted to translate these findings into clinical applications and to develop optimized treatment protocols for OSF patients.
